# A Comparative Study of Automated Pulsed Bolus Versus Continuous Basal Infusion on Distribution of Dye in the Paravertebral Space in a Cadaver

**DOI:** 10.7759/cureus.4958

**Published:** 2019-06-20

**Authors:** Steven R Clendenen, Elird Bojaxhi

**Affiliations:** 1 Anesthesiology, Mayo Clinic, Jacksonville, USA

**Keywords:** paravertebral block, intermittent pulsed dosing, thoracic surgery

## Abstract

Adequate pain control following thoracic surgery is important to enhance post-operative recovery. Paravertebral catheters have been reported to have a variety of clinical applications, including the blunting of surgical pain, lessening the need for opioids, and improvement in post-operative ventilation. The spread of local anesthesia to multiple paravertebral spaces is needed to establish an effective block. We have determined that the spread of contrast dye by a catheter in the thoracic paravertebral space is greater in a programmed intermittent bolus than in a continuous basal infusion.

## Introduction

A variety of methods have been used for the delivery of local anesthetics by catheters for a continuous nerve block, including elastomer and electronic pumps. Some of the newer electronic models have the capability of programmed intermittent bolus (PIB) in contrast to continuous basal infusion (CI). This feature can potentially improve the efficacy of a nerve block by expanding the spread of local anesthetic in the perineural space. Catheters in the thoracic paravertebral space are used to deliver a continuous infusion of local anesthetics in order to provide prolonged analgesia following chest surgery [1-2]. It has also been noted that when a local anesthetic is delivered as a bolus in the paravertebral space, it can potentially provide analgesia up to six dermatomes beyond the initial injection [3]. Therefore, the efficacy of the block for postoperative analgesia is also dependent on the spread of local anesthesia to multiple levels of the paravertebral space. The goal of this cadaveric study is to investigate the spread of equal volume of contrast dye in the paravertebral space between PIB and CI mode of deliverance.

## Technical report

**Figure 1:**
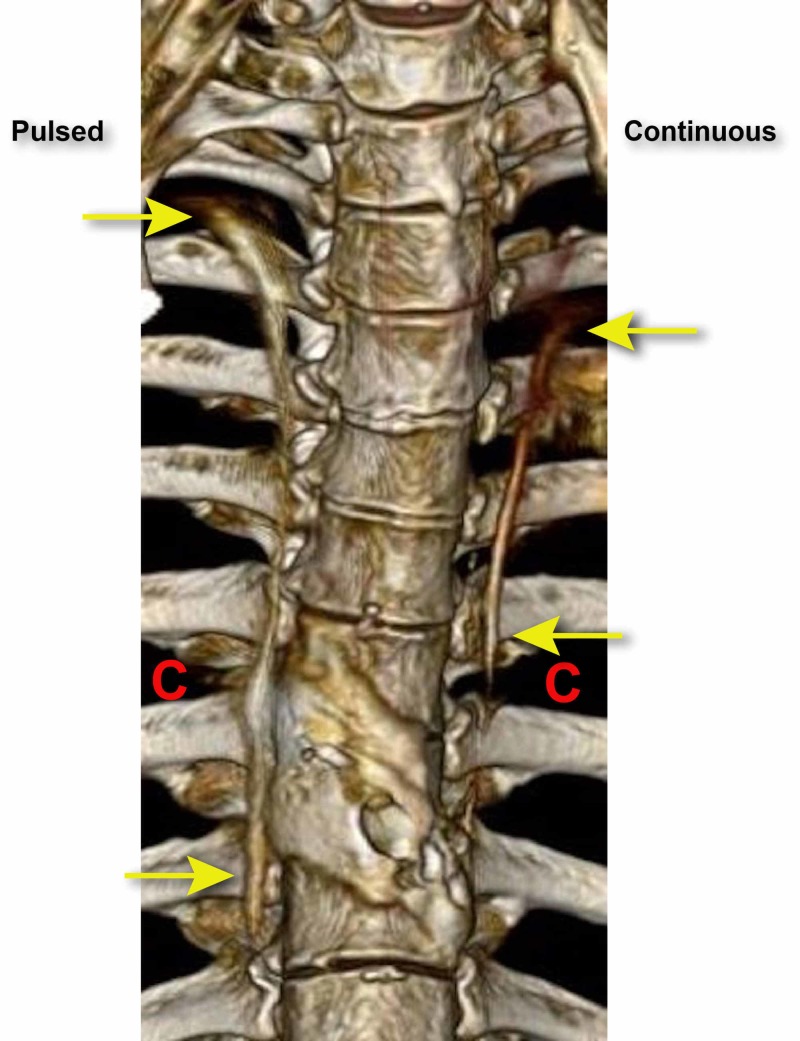
The distribution and spread of the dye after 10 mls infused. The red Cs indicate the location of paravertebral catheters. The yellow arrows indicate the superior and inferior spread of dye.

**Figure 2:**
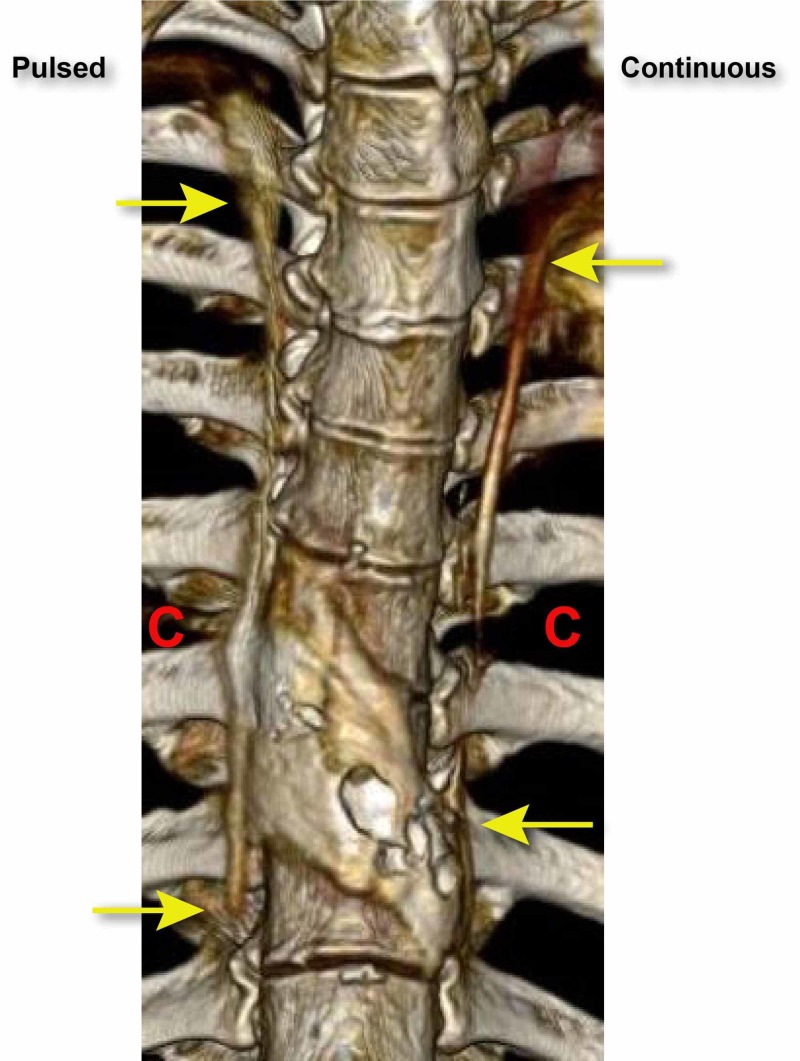
The distribution and spread of the dye after 15 ml infused. The red Cs indicate the location of paravertebral catheters. The yellow arrows indicate the superior and inferior spread of dye.

**Figure 3:**
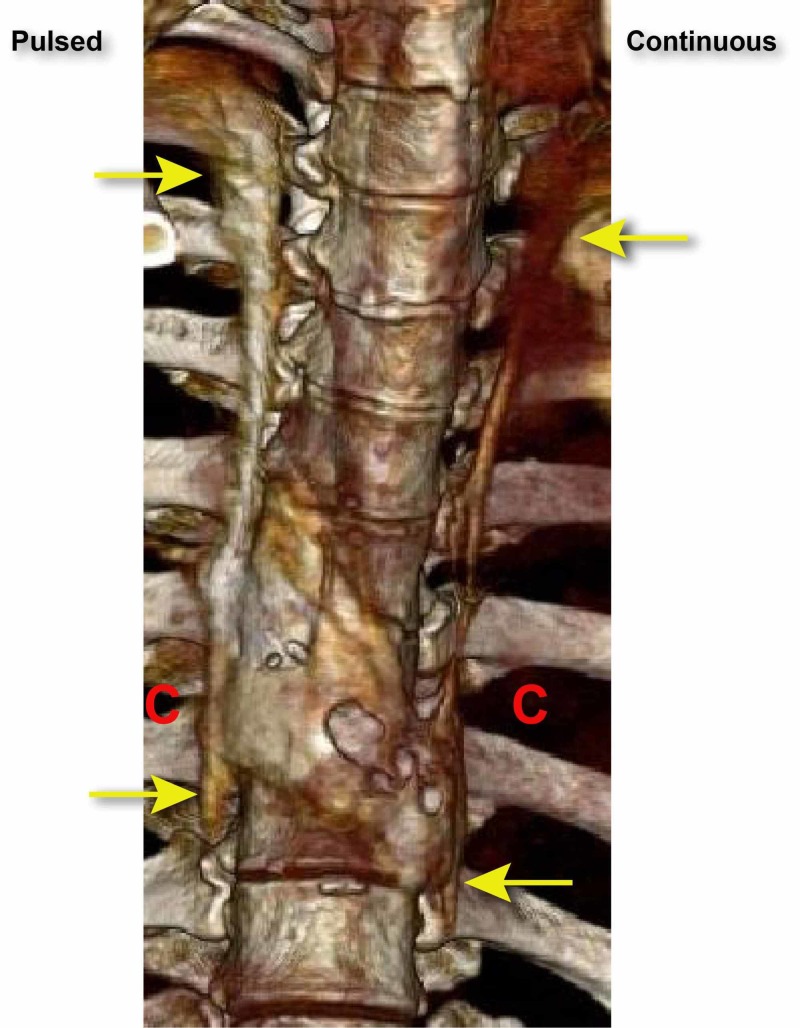
The distribution and spread of the dye after 20 ml infused. The red Cs indicate the location of paravertebral catheters. The yellow arrows indicate the superior and inferior spread of dye.

A fresh cadaver was placed in the prone position and bilateral paravertebral catheters were placed at the level of the fifth thoracic spine. The spinous process was palpated and 2.5 centimeters lateral to midline an 18 Ga x 9 cm. Tuohy needle (B. Braun Medical, Bethlehem, PA) was inserted to the transverse process. The needle was then advanced 1 cm below the transverse process with a loss of resistance technique into the paravertebral space. Soft tip 20 Ga catheters with three lateral side ports (Perifix Epidural Catheter, B. Braun Medical, Bethlehem, PA) were inserted 3 cm beyond the needle tip and secured in place. The cadaver was transported and placed on a CT scanner table and remained stationary after the infusions were started. The two catheters were attached to an infusion of iohexol dye (Omnipaque, GE Healthcare Ireland, Cork, Ireland) diluted 1:10. The catheter on the left side was assigned to a CI of 5 mL/h and the catheter on the right was assigned to the PIB with an automated 5 ml hourly boluses (PIB-PCA, Ambit, Summit Medical Products, Sandy, Utah). A volume rendering CT scan at 0.5 mm slice was obtained after 10 ml (Figure 1), 15 ml (Figure 2), and 20 ml of dye was infused (Figure 3).

No leakage of dye was observed from any of the catheters. At each interval of dosing at 10, 15, and 20 ml, there was a greater rostral spread and circumferential expansion of the dye in the PIB catheter when compared to the CI catheter.

## Discussion

Our study demonstrated that a greater spread of dye was observed in the paravertebral space with PIB when compared to an equal infused volume via a CI. The pump is a constant volume pump which adjusts its delivery pressure to administer the programmed volume within a set time interval. A CI of a rate of 5 ml/hr infuses at 1 ml even 12 minutes. In comparison, PIB set at 5 ml/hr to be administered at the end of each hour interval will deliver the dose at 210 ml/hr (as per manufactures specifications), thus 5 ml is infused in 86 seconds. Therefore, the rapid infusion of the local anesthetic in the paravertebral space will likely result in greater local anesthetic spread along the paravertebral gutter, thus covering more thoracic nerve roots. Based on these results, it can be extrapolated that PIB in the clinical setting will improve the efficacy of the block when compared to CI, while maintaining the same hourly dose. 

However, the clinical evidence to support this hypothesis is currently inconclusive. Chen et al. demonstrated that hourly PIB in the thoracic paravertebral catheters for unilateral video-assisted lung resection was superior to CI by decreasing the need for additional patient controlled doses of local anesthesia, reduced pain scores, and improved patient satisfaction [4]. On the contrary, a study by Català et al. demonstrated that PIB provided inferior pain control in comparison to a CI in a thoracic PVB catheter, but the pulsed interval was six hours [5]. Other studies did not show a significant difference in pulsed dosing versus continuous infusions in targeting the femoral and adductor canal blocks [6-7]. A current literature review on comparative effectiveness by Jagannathan et al. showed no advantages to intermittent pulsed dosing over continuous basal infusion [8]. However, the review also illustrates the limited number of studies on the topic which comprised mostly small samples, with the methods having a great degree of heterogeneity. Furthermore, the mechanism of action and perineural anatomy differs greatly between different types of blocks. It is important to consider that PIB may play a significant role in only specific type of blocks, such as fascial plane blocks or PVBs, which are more dependent on a broad spread of local anesthetics.

## Conclusions

The programmed intermittent thoracic paravertebral bolus demonstrated a greater spread of radiopaque dye in comparison to the continuous infusion. Further prospective clinical trials are needed to prove if there is greater efficacy of intermittent pulsed dosing.
